# Barriers, Facilitators and Preferences for HIV Testing Services among Adolescent Girls (15–19) and Young Women (20–24) in Rakai District, Central Uganda

**DOI:** 10.21203/rs.3.rs-6744863/v1

**Published:** 2025-07-14

**Authors:** Christine Muhumuza, Caitlin E. Kennedy, Mayanja M. Kajumba, Joseph Kagaayi, Joan Nakayaga Kalyango, Joaniter I Nankabirwa, Bridget Nagawa Tamale, Lynn Atuyambe. Muhimbura

**Affiliations:** Makerere University School of Public Health; Johns Hopkins Bloomberg School of Public Health; Makerere University School of Psychology; Makerere University School of Public Health; Makerere University Kampala; Makerere University School of Public Health; Makerere University School of Public Health; Makerere University School of Public Health

**Keywords:** HIV Testing Services, Adolescent Girls and Young Women, Uganda, Rakai, Qualitative Study

## Abstract

**Background:**

HIV remains a significant global public health challenge, disproportionately affecting adolescent girls and young women (AGYW). Uganda has adopted different strategies of HIV Testing Services (HTS) to improve access and utilization, but the uptake of these services by AGYW is still low. This study explored barriers, facilitators, and preferences for HTS among AGYW in Rakai district, Central, Uganda.

**Methods:**

This qualitative study employed in-depth interviews with 24 purposively selected AGYW aged 15–24 years who had used HTS at least once in the past year. Participants were drawn from diverse backgrounds based on age, residence (fishing or mainland communities), schooling, employment, and marital status. Data were analyzed thematically using both inductive and deductive approaches in Atlas.ti software.

**Results:**

Key barriers to HTS uptake varied by age and residency. Adolescent girls aged 15–19 years, especially those in school, expressed strong fear of blood-based testing and discomfort with invasive procedures, while young women aged 20–24 years emphasized long waiting times, provider attitudes, and confidentiality concerns, particularly in public facilities. AGYW from fishing communities reported heightened stigma and privacy challenges in public facilities, making them more reliant on discreet mobile and outreach services. In contrast, mainland residents favored private facilities for their efficiency. Facilitators across all groups included mobile HTS and community outreaches, which improved accessibility and reduced logistical barriers to utilization. Younger adolescents preferred saliva-based, less invasive tests, while older participants favored facility-based testing for the professional support it offered. Preferences also varied across provider characteristics: married and older AGYW favored experienced adult providers, while younger participants, particularly in mainland areas, expressed comfort with male providers seen as more empathetic.

**Conclusion:**

HTS strategies should offer a diverse range of service delivery options that reflect the varied preferences and needs of AGYW by age and residency. Expanding mobile testing, offering less invasive options, ensuring confidentiality, and improving provider interactions are critical for increasing HTS uptake in high-risk settings like Rakai.

## Background

Adolescent girls and young women (AGYW), aged 15–24, continue to bear a disproportionate burden of new HIV infections in sub-Saharan Africa (1). In 2023, AGYW were more than three times as likely to acquire HIV as their male peers, largely due to gender inequalities, poverty, and social vulnerability (1–3). In Uganda, recent estimates indicate that AGYW accounted for nearly 79% (15,000 out of 19,000) of all new infections among young people in 2022 (4). To close the gaps in HIV testing coverage, the World Health Organization (WHO) recommends expanding HTS that includes self-testing for the initiation, integration with pre-exposure prophylaxis (PrEP), promoting testing through sexual and social networks (5). Despite these global and national efforts to scale up HTS, uptake among AGYW remains low due to persistent gender inequalities, stigma, and limited youth-friendly services (1, 6, 7). While Uganda has implemented a range of strategies to expand HTS including self-testing, peer-led approaches, and integration into reproductive health services, (8–11) little is known about how AGYW perceive and engage with these different testing options. Few studies in Uganda and East Africa have specifically examined AGYW’s preferences for HIV testing modalities, and those that exist often focus broadly on youth or key populations without disaggregating findings for AGYW. This lack of targeted evidence represents a critical gap, as understanding barriers, facilitators and preferences is essential to designing interventions that resonate with AGYW and improve HTS uptake.

We explored barriers, facilitators and preferences for HTS among AGYW in Rakai District, Uganda. The findings aim to generate context-specific evidence that can guide policy, inform program design, and enhance the accessibility and acceptability of HTS for AGYW.

## Methods

### Study design

This qualitative study, conducted as part of a larger exploratory sequential mixed-methods study, used in-depth interviews to explore barriers, facilitators, and preferences related to HTS among AGYW (12).

### Study setting

Rakai is a district in central Uganda, located approximately 150 km southwest of Kampala, with an HIV prevalence estimated at 12.2%, significantly higher than the national average (13). This disproportionately high burden of HIV makes Rakai a critical setting for research focused on adolescent girls and young women (AGYW), a group already at elevated risk of infection.

The study was conducted among AGYW enrolled in the Rakai Community Cohort Study (RCCS). The RCCS is a longitudinal population-based cohort that has been ongoing since 1994 and includes approximately 19,000 individuals aged 15–49 years (14). Conducted by the Rakai Health Sciences Program, the RCCS aims to provide vital insights into HIV epidemiology and health services utilization through periodic surveys conducted every 18 months (14). In addition to study-based activities, the Rakai Health Sciences Program offers HTS to all cohort participants and links all HIV-positive individuals to ART services.

### Study population and sampling

This study included AGYW who are enrolled in the RCCS cohort. Participants were purposively included if they had received HTS, resided in the Rakai, and were willing to provide informed consent. Participants were excluded if they were mentally unstable, ill, or unavailable at the time of data collection. With the aid of community mobilizers, including the Village Health Team members and local council members, eligible participants were identified and interviewed.

Purposive sampling was used to select 24 AGYW with prior HTS experience; participants were selected based on socio-demographic characteristics, including age, marital status, type of residence (fishing community or mainland), and occupation to ensure diverse representation. Inclusion criteria included being females aged 15–24 years, having utilized HTS at least once in the past year, and being willing to share their experiences. To reduce stigma and ensure confidentiality, a trained female community mobilizer identified eligible AGYW through health facilities and community networks, creating a safe and trusting environment for participation.

### Data collection, management, and analysis

IDIs were conducted using structured IDI guides developed specifically for this study, which was informed by existing literature on HTS and adolescent health (15–17). Interviews were conducted in Luganda, lasted between 60 and 90 minutes, and were audio-recorded to allow interviewers to focus on the conversation without missing key details. All interviews were conducted in private settings to ensure confidentiality and encourage candid responses. While the HIV status of participants remained confidential, some chose to disclose it during the discussions.

Data were transcribed verbatim in Luganda and translated from Luganda to English by trained qualitative research assistants. Data analysis was conducted using Atlas.ti 8 software, applying both inductive and deductive thematic analysis approaches (18). Initially, transcripts were reviewed multiple times to ensure a thorough understanding, followed by the development of codes and codebook definitions aligned with study objectives while incorporating emerging themes. Two qualitative analysts reviewed and agreed on the codebook before coding the transcripts.

During analysis, paraphrases or labels (codes) were applied to significant passages, categorizing them into substantive values. Inductive coding was first employed to identify emergent themes directly from the data, ensuring participants’ perspectives shaped the analysis. This was followed by deductive coding to align identified themes with the study objectives, providing a structured understanding of factors influencing HTS uptake and preferences. To validate findings, a random selection of coded passages was reviewed by an external person who independently developed codes. A comparison of these codes with those generated by the research team relatively matched those of the other analysts and this helped ensure the validity of the coding process.

## Results

### Characteristics of the study population

A total of 24 AGYW aged 15–24 years were interviewed. Half aged 15–19, and half 20–24. Thirteen (13) resided in fishing communities, and eleven in mainland areas. Six (6) were in school, six (6) were married, and twenty (20) were in informal employment. Table 1 provides a summary of the demographic characteristics of the 24 AGYW who participated in the IDIs.

### Barriers, facilitators, and preferences for HTS

**Figure 1:**
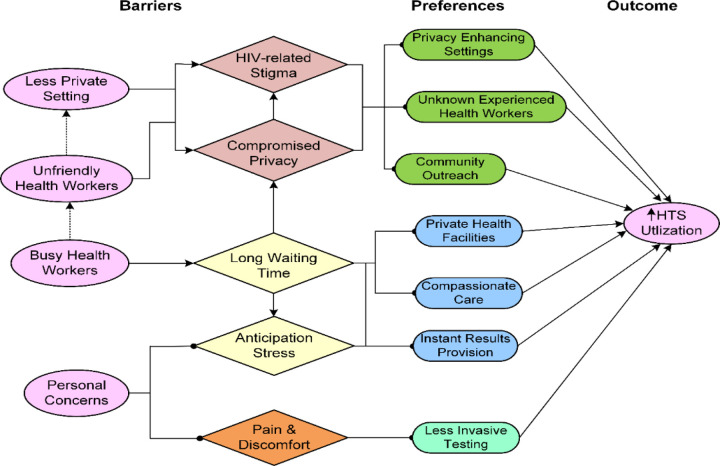
The visual display of the study results

This section presents key findings across three thematic areas –barriers, facilitators, and preferences for HTS while highlighting variations by key demographic characteristics. Figure 1 is the visual display of the summary of results over all groups of participants.

### Fear of Pain from Blood Tests

Concerns about the pain associated with blood tests emerged as a significant barrier to the utilization of HIV testing services, particularly among younger participants (aged 15–19). These girls expressed a strong dislike of invasive testing methods that include needle pricks and drawing of blood, which cause discomfort and pain, describing the process as both physically and emotionally distressing. Majority of the participants from both mainland and fishing communities highlighted that pain discouraged them from pursuing testing.

“…my cousin has never tested because of that…..even my friends fear the needle… I think in the laboratory, the finger-prick for HIV blood test should be changed to have the tests with the saliva and urine, if it is possible.” (D5: ID04:15–19_ Fishing Community)

### Long Waiting Times at Public Health Facilities

This barrier was common across all groups. Participants highlighted a struggle to balance waiting times with school or work schedules. Nearly all the working and in-school participants reported that public health facilities were often seen as understaffed and inefficient, leading to significant service delays. These participants shared that the extended waiting times often deterred them from utilizing services, especially when these delays conflicted with their school time or personal schedules. Most older participants (aged 20–24) emphasized the importance of improving staffing levels, streamlining services, and introducing youth-friendly policies to reduce waiting times, which would make health facilities more appealing and accessible to them and their colleagues.

“The place where blood is tested is the same place where people who have come for HIV viral load go, and other HIV services, also, the waiting time is long. You know we girls lose interest in these things so fast, so many girls leave the facility without getting the service easily.” (D12: (Unmarried_20–24).

### Negative Attitudes of Health Workers and Confidentiality Concerns

A few participants, particularly those aged 20–24, from fishing communities and those who were married, reported experiencing negative interactions with health workers, which they identified as a significant barrier to utilizing HTS. These interactions often included rudeness, dismissive behavior, and a lack of attention to maintaining patient privacy. Participants, mainly those who were married, expressed feeling undervalued and disrespected during their visits, which contributed to a sense of mistrust and reluctance to seek even other health care services. Most participants from the fishing community highlighted the lack of privacy, as it exacerbated feelings of discomfort and vulnerability, particularly in sensitive situations such as receiving test results or discussing personal health concerns. These participants emphasized that confidentiality is not only a matter of professional ethics but also a critical factor influencing their willingness to engage with healthcare services even beyond HTS. Instances where confidentiality was breached or perceived to be at risk deterred these AGYW from returning to facilities, including receiving test results after taking the test. These participants also highlighted the importance of respectful and calm interactions, noting that the approach and attitude of health workers significantly affected their overall experience. For many, the behavior of health workers during initial visits played a key role in determining whether they would return to the facility for subsequent services, including waiting to receive test results.

“I want a health worker who is not rude because when you become rude, I may not come back the following day to the facility or to wait for the test results... We prefer health workers that are calm so that you can reach the facility and joke with them.” (D3: 20–24 Married).

### Facilitators of HTS Uptake

#### Mobile HTS and Community Outreach

Mobile HTS and community outreach services were widely recognized by participants as encouraging AGYW to utilize HIV testing. These services were particularly appreciated for their ability to bring healthcare closer to individuals, reducing the need for travel to distant health facilities. Participants from fishing communities and working individuals highlighted the convenience of mobile testing units stationed at workplaces, community centers, or other accessible locations. For those engaged in fishing, whose demanding schedules often limit their ability to visit health facilities, mobile testing provided an opportunity to receive timely and essential health services without disrupting their daily activities. Similarly, participants in inland communities noted that outreach initiatives helped mitigate logistical barriers such as transportation costs, long distances to health facilities, and the need to take time off work or school.

Community outreach efforts were also praised for their ability to create a supportive and inclusive environment that encouraged AGYW to participate in health services. Participants emphasized that these programs not only provided HIV testing but also included educational sessions that raised awareness about HIV prevention, treatment, and the importance of early testing. These initiatives were seen as empowering, helping to reduce stigma associated with HIV testing and creating a sense of collective responsibility within the community to address public health challenges. Mobile testing services were highlighted mainly by in-school adolescents for their flexibility and adaptability, offering services during non-traditional hours and in convenient locations. This approach made testing more accessible to these AGYW with limited time or financial resources, ultimately contributing to increased uptake of HTS.

“It is easy for adolescent girls to test with health workers from the Rakai Project because they test at the adolescents’ workplaces. They collect a blood sample from there and then go back to work.” (D3: 20–24_Mainland)

### HTS Preferences

#### Facility-Based Testing vs. Self-Testing

Majority of participants expressed a strong preference for facility-based HIV testing over self-testing, citing the professional support and guidance provided by healthcare workers as a primary advantage. Facility-based testing was regarded as more reliable and reassuring, as participants valued the opportunity to receive immediate counseling, personalized care, and accurate interpretation of test results. All adolescent girls highlighted that this support system played a crucial role in helping them navigate their healthy journeys with confidence and clarity. Younger participants (15–19) appreciated the guidance during stressful first-time testing, while older AGYW (20–24) feared misinterpreting results and emphasized the value of face-to-face interaction. Some of the participants emphasized that the counseling offered at health facilities was particularly beneficial. They noted that healthcare workers provided critical information on HIV prevention and treatment, enabling them to make informed decisions about their health. The availability of healthcare professionals also ensured that participants felt emotionally supported during what they called a stressful process, particularly for those undergoing testing for the first time or who feared a positive result.

“Testing at health facilities is better because the health workers will be aware of your status. A person may self-test and feel embarrassed to go and look for treatment at the health facility.” (D13: _20–24_Fishing Community)

Self-testing, on the other hand, was met with caution and doubt. Majority of the participants, especially the young women, raised concerns about the lack of professional oversight and the potential for misinterpretation of results using self-testing. They expressed apprehension about confidentiality, which comes with worrying that self-testing kits could unintentionally expose their health status to others, particularly in shared living environments like fishing communities. Moreover, the absence of immediate professional guidance raised fears that individuals might not seek appropriate follow-up care if they tested positive. Majority of the participants also highlighted the social and psychological dimensions of facility-based testing. The structured environment of health facilities was seen as promoting accountability and encouraging individuals to take proactive steps toward managing their health. By contrast, the majority of the married women indicated that the isolation associated with self-testing was viewed as a potential barrier to emotional well-being and effective health supervision, particularly for those who lacked a strong support system.

#### Less Invasive Testing Methods

A significant preference for less invasive HIV testing methods emerged particularly younger adolescents (15–19 years), residing in both mainland and fishing communities. They strongly preferred saliva-based tests over traditional blood-based methods due to fear of needle pricks and discomfort. This age group expressed anxiety around invasive procedures and viewed saliva tests as a pain-free and more approachable alternative, especially suitable for school settings and first-time testers. Majority of the older AGYW (20–24 years), while generally more accustomed to medical procedures, also supported the introduction of non-invasive options, particularly for improving HTS utilization in community and workplace outreach programs. Across both residency groups, saliva-based testing was perceived as a practical and youth-friendly solution that could increase overall HTS uptake. Saliva-based testing was suggested as a less painful and more user-friendly alternative, offering a viable solution to the barriers associated with traditional methods. Nearly all participants noted that the non-invasive nature of saliva-based tests could not only reduce fear and discomfort but also encourage their peers to consider testing. They emphasized that saliva-based methods would be more accessible and less intimidating, especially for first-time testers or individuals with heightened sensitivity to needles.

Nearly all older AGYW (20–24 years) and few of the adolescent girls (15–19 years) pointed out the psychological benefits of less invasive testing. For many, the idea of avoiding pain and physical discomfort made the process feel less daunting, fostering a more positive perception of HIV testing. This, in turn, could lead to increased uptake of testing services, particularly among AGYW who may be apprehensive about healthcare procedures. Moreover, saliva-based testing was highlighted as a practical alternative to be used alongside community-based or mobile testing initiatives. Participants suggested that introducing non-invasive options could broaden access and utilization of testing in schools, workplaces, and other community settings, where logistical and emotional barriers often limit participation. The ease of administering saliva tests was highlighted as a factor that could make testing campaigns more inclusive and efficient.

“If it is possible, saliva should be used. For instance, they bring testing kits where you put your saliva and get tested immediately. An adolescent girl may hesitate to go to the health facility with fear of getting pricked and experiencing pain. Based on that, she may decide not to take on the HIV testing service...” (D5:15–19 Mainland).

#### Experienced Healthcare Providers

The majority of the married AGYW, primarily from both mainland and fishing communities, favored older and more experienced healthcare providers, citing their professionalism, discretion, and ability to handle sensitive health concerns with maturity and empathy. Equally, several adolescent girls (15–19 years), especially those from mainland areas and in-school settings, reported a preference for male service providers, describing them as more patient, respectful, and gentle compared to their female counterparts, who were sometimes perceived as judgmental or harsh towards them because of their age.

“I feel like a young service provider doesn’t have enough experience. I would prefer an adult person who has had the same experience. I was once abused by the nurse while in the que and I almost left but Jakie (not real name) my friend convinced me to stay…” (D14: 15–19_Married)

#### Private and Community-Based Services

A strong preference for private health facilities and community-based testing services emerged among participants, particularly those who were employed. Private health facilities were consistently praised for their efficiency, with participants highlighting the prompt delivery of services as a major advantage over public health facilities. Many employed respondents noted that private facilities offered flexible scheduling and shorter waiting times, which better accommodated their busy work schedules. This efficiency allowed them to prioritize their health without disrupting their professional obligations.

Community HTS outreach programs were similarly appreciated for their convenience and accessibility. Some of the participants noted that these programs brought health services closer to their homes or workplaces, eliminating the need for time-consuming visits to traditional health facilities. Community-based testing was particularly advantageous for individuals in rural or underserved areas, as it reduced logistical barriers such as transportation costs and long travel distances. The informal and familiar setting of outreach programs also were highlighted to alleviate the anxiety often associated with visiting formal health facilities, encouraging greater participation and utilization of HTS.

However, a contrasting perspective was highlighted by some unemployed participants, who expressed concerns about the cost associated with private health facilities. While they recognized the advantages of efficiency and quality, they noted that high service fees often posed a significant barrier to utilizing HTS and other services. For many, the financial burden of private healthcare made it an impractical option, limiting their ability to utilize HTS consistently. These participants emphasized the need for affordable and subsidized testing services offered by private health facilities to ensure equitable access and utilization across all socioeconomic groups.

“I prefer to visit the private health facility and get attended to as early as possible. At the private health facility, you just explain to the healthcare provider, and they start attending to you immediately.” (D14: _15–19_ Fishing Community).

Lastly, private facilities were highlighted for their efficiency and convenience for employed AGYW, while community-based programs provide accessible solutions for geographically and logistically constrained populations of AGYW.

## Discussion

This study identified key barriers, facilitators, and preferences shaping HTS uptake in Rakai District; an area with historically high HIV prevalence. Kasensero, for instance, a fishing community in Rakai has an HIV prevalence of 44.3% and extending to 74.5% (almost 10-fold the national figures) among female bar workers, who are often AGYW (19, 20). The high HIV prevalence in Rakai not only reflects the high rates of transmission but also amplifies the potential public health impact and the need for targeted interventions in this setting. Among such interventions is targeted HTS for AGYW, who remain at elevated risk of infection due to social vulnerability, early sexual debut, limited negotiating power in relationships, and restricted access to youth-friendly services (21, 22). However, despite efforts to improve HTS uptake among AGYW, as an entry point to care and a critical component of the broader HIV prevention strategy (23), its successful implementation is still a major challenge, with more than 54 to 60% of HIV positive AGYW still unaware of their HIV status (24).

This study identified several significant barriers to utilizing HTS, including concerns about pain from blood tests, long waiting times, privacy issues, and negative interactions with healthcare workers. This consistent with a previous Malawian study, which found that participants were less likely to utilize HIV testing services if they perceived the services to lack privacy, to be offered at inconvenient times, to involve long distances or waiting time, or having rude providers (25). These barriers reflect the costs and inconveniences associated with HIV testing that discourage AGYW from seeking these services. Existing literature consistently highlights similar barriers to HIV testing uptake. For instance, the fear of pain from blood tests, long waiting times, and privacy issues are well-documented HTS impediments in low- and middle-income countries (26–29). Additionally, the behavior and attitude of healthcare workers play a crucial role in influencing patients’ willingness to seek care (30). Negative experiences with healthcare providers can significantly deter AGYW from accessing HTS.

This study found that the barriers and facilitators of HTS uptake among AGYW could be influenced by age, residency, and service delivery modalities. For instance, younger adolescents (15–19) feared pain from blood tests and preferred non-invasive methods like saliva-based testing, while older AGYW (20–24) prioritized confidentiality, efficiency, and respectful provider interactions. AGYW from fishing communities faced heightened stigma and privacy concerns, favoring community-based outreach, whereas those in mainland areas preferred private facilities for their convenience. Overall, the privacy concerns highlight the critical need for confidential and respectful service delivery.

Across groups, there was a strong preference for facility-based testing with professional counseling offered by experienced, empathetic healthcare providers. Interesting, the AGYW in our study expressed preferences for experienced, adult, and often male healthcare providers, as well as privacy and confidentiality in testing services. This preference can be attributed to the serious nature with which AGYW view an HIV diagnosis, necessitating the involvement of healthcare providers perceived as experienced and trustworthy. Adult and male healthcare workers are often seen as more caring and competent, reflecting AGYW’s desire for high-quality care in a private and confidential setting (31–33). This finding aligns with other studies where patients express a preference for healthcare providers they perceive as more competent and capable of maintaining confidentiality (34–36). The preference for male providers can also be linked to cultural and social norms prevalent within specific communities. To enhance the perceived quality and acceptability of HTS, it is essential to train and deploy experienced healthcare workers who can maintain confidentiality and where possible, to consider patient preferences regarding the gender of providers.

Addressing the barriers to HTS uptake requires a multifaceted approach that puts AGYW’s experiences and preferences into consideration. For instance, in this study, the AGYW preferred non-invasive HIV testing techniques, such as saliva-based or urine-based tests, which can mitigate concerns about pain and increase HTS uptake. Evidence suggests that the use of these non-invasive techniques not only addresses the concerns about pain, but the privacy concerns, through the reduced need for visiting health facilities (37, 38). Studies in SSA countries such as Malawi, Zimbabwe, Mozambique, and Kenya (39–41), as well as China and Australia (42–45), have shown that less invasive methods, such as oral fluid tests, are generally more acceptable to individuals who fear needles or the sight of blood. Apoola and Brunt (44), for instance, reported that an oral swab test in the community for blood-borne virus testing leads to an increase in the number of young high-risk people tested for blood-borne infections. In contrast, other similar studies in Nigeria (46), India (47), and Tanzania (48) reported a preference for blood-based HIV self-testing compared to oral HIV self-testing due to a lack of familiarity and concerns about accuracy with oral HIV self-testing. Therefore, introducing both invasive and non-invasive HIV self-testing methods can guarantee privacy and increase the uptake of HTS among AGYW.

Ensuring privacy in testing settings is crucial for encouraging more AGYW to seek testing, thereby improving HIV testing uptake and early diagnosis. Indeed, previous research shows that private healthcare settings often deliver better patient experiences through shorter wait times and more personalized care (49–52). Additionally, community-based testing has been shown to significantly improve access and uptake of health services, particularly in hard-to-reach populations (23, 53, 54). To enhance the accessibility and acceptability of HIV testing among AGYW, it is crucial to strengthen private-public partnerships and expand community-based testing programs. Incorporating these strategies into national HIV prevention and testing campaigns can address the logistical and experiential barriers that currently hinder HTS uptake among AGYW.

Reducing waiting times through improved healthcare facility management, increased staffing, and utilization of rapid testing can enhance the overall AGYW experience and minimize the fear and anxiety associated with the possibility of being seen at the stigma-associated HIV service clinics. In this study, AGYW from fishing communities reported more pronounced privacy concerns, probably due to close-knit social networks where breaches of confidentiality can lead to stigma. This could be mitigated through the use of community-based or mobile testing services, as well as ensuring privacy in HIV testing settings. Mobile and community-based services are not only valuable as a privacy-enhancing strategy but could also be pivotal in reducing transportation costs and increasing convenience; thereby contributing to increased HTS uptake. Thus, mobile HTS and community outreach are highly effective HTS strategies for addressing logistical challenges and making testing more accessible. For instance, mobile testing services provided at workplaces or within communities significantly reduce barriers, allowing AGYW to access HTS without disrupting their daily activities. The effectiveness of mobile testing and community outreach in increasing HIV testing is well-documented. For instance, studies in Kenya (55), Lesotho (56), and Zimbabwe (57) have shown that bringing services closer to where people live and work significantly improves access and uptake, particularly among populations with limited access to healthcare facilities. Given these findings, expanding mobile HTS and community outreach programs may support increased reach and uptake of HTS among AGYW. These initiatives should be a priority in public health planning to ensure broader access and convenience, ultimately contributing to better health outcomes for this population.

However, in contrast to those in fishing communities, AGYW in mainland communities, particularly those in school or employed, emphasized the value of flexible scheduling and efficient service delivery, which are often best met through private or school-based HTS programs. AGYW’s preference for facility-based testing over self-testing emphasizes the importance of self-efficacy in the HIV testing decision-making process. These AGYW favor the structured environment of health facilities where they receive comprehensive counseling and support, reflecting a lack of confidence in self-testing and a recognition of the benefits of professional support. Other studies indicate that self-efficacy significantly influences the acceptance of self-testing. Individuals with higher confidence in their ability to manage health outcomes are more likely to opt for self-testing (28, 58, 59). On the other hand, concerns about the correct use of self-tests and the availability of follow-up care make facility-based testing more appealing. To increase the acceptability of self-testing, it is essential to enhance self-efficacy through targeted education and support programs. Nevertheless, maintaining robust facility-based testing services with professional support is crucial to ensure comprehensive care and appropriate follow-up strategies. This dual approach can help address the needs and preferences of AGYW, ensuring they receive the necessary support regardless of the testing strategy they choose.

However, in this study, the AGYW indicated that the utilization of health facility-based services is affected by negative behavior and attitudes of health workers. These negative behaviors and attitudes of health workers could be addressed through training them in patient-centered care, which is very essential in making services more user-friendly and encouraging AGYW to seek testing. Furthermore, health system reforms focusing on addressing the barriers to HTS utilization, the service users’ preferences, and enhancing the users’ experience are key for improving the uptake of HIV testing among AGYW.

Interestingly, our findings highlight differences in experiences and needs across age groups and residency. For instance, whereas younger adolescents (15–19 years) were more concerned about HIV testing-related physical and setting discomfort, while older AGYW (20–24 years) were more concerned with issues of efficiency, confidentiality, and respectful provider interactions. These differences reflect varying stages of maturity, autonomy, and exposure to health systems, underscoring the need for age-appropriate HIV testing strategies. Indeed, previous studies found such age-related discrepancies in preferences can affect HTS uptake, with younger AGYW (15–19) being less likely to utilize the services or pick results after HIV testing, compared to the older AGYW (20–24 years) (8, 24, 60).

### Limitations of the study

This study was conducted within the RCCS, meaning the findings reflect the experiences of AGYW within this specific setting and may not fully represent those in other regions with different healthcare systems and HIV prevalence. Since the study relied on individual interviews, participants may have shared their experiences in ways that align with social norms or expectations, potentially shaping how they described their engagement with HTS. Additionally, the purposive sampling approach, while useful for capturing diverse perspectives, may have excluded AGYW with differing experiences or viewpoints on HTS. Although qualitative design allowed for rich, in-depth exploration, it may not provide a complete picture of broader trends in HTS uptake. Future research would benefit from a mixed-methods approach, combining qualitative insights with quantitative data to deepen understanding and ensure a more comprehensive perspective on the factors influencing HTS preferences among AGYW.

## Conclusion

The barriers to HTS utilization among AGYW included concerns about pain from blood draw during the tests, long waiting times, privacy issues, and negative interactions with healthcare workers. Addressing the identified barriers, with particular attention to the users’ preferences, can create a more supportive and accessible environment for HIV testing; ultimately contributing to better health outcomes for this vulnerable population. We, for instance, found that AGYW preferred less invasive HTS, provided by experienced, adult, and often male healthcare providers, with guaranteed privacy. Tailoring HIV testing services to the unique needs of AGYW based on their age, setting, and lived experiences can significantly increase uptake and improve health outcomes. Through addressing key barriers such as fear of pain, stigma, and inefficient service delivery, and by building supportive, youth-friendly environments, Uganda can make meaningful progress in reducing HIV risk among AGYW, who are one of its most vulnerable populations. However, Uganda’s HIV testing and prevention programs mainly rely on donor support, and the recent shifts in U.S. government funding priorities, particularly the reduction of support for HIV services under global health assistance policy changes, risk-undermining the progress made in AGYW-focused HIV programming. These policy shifts have led to reduced funding for community-based outreach, youth-friendly services, and HIV programming including HIV testing, which disproportionately affect AGYW’s access to holistic and stigma-free care. Despite these challenges, our findings point to actionable recommendations.

### Recommendations

To improve HTS uptake among AGYW, policymakers and healthcare providers should offer a diverse range of service delivery options that reflect the varied preferences and needs of AGYW by age and residency. For younger adolescents, introducing non-invasive methods like saliva-based tests, expanding school-based and mobile outreach, and ensuring adolescent-friendly care are critical. Older AGYW would benefit from efficient, facility-based services with professional counseling and flexible scheduling. In fishing communities, mobile outreach and strong confidentiality measures are essential to overcome stigma and access barriers. For mainland and urban populations, strengthening public-private partnerships and workplace-based testing can enhance accessibility and utilization. Across all settings, healthcare providers should be trained in good quality health care approaches, youth-friendly, respectful, and confidential service delivery. Programs should also be safeguarded from policy disruptions through sustainable funding and integrated sexual and reproductive health services. Overall, addressing these age and context-specific barriers and preferences is vital for strengthening HTS uptake and improving health outcomes for AGYW in high-prevalence settings like Rakai.

## Supplementary Material

1

## Figures and Tables

**Table 1:** Participants’ Sociodemographic Characteristics

Data Collection	Variables	Categories	N
In-depth Interviews (n = 24)	Age	15–19	12
		20–24	12
	Education status	In-school	6
		Out of School	18
	Marital Status	Single	18
		Married	6
	Occupation	Employed	20
		Student	4
		Unemployed	0
	Residence	Fishing Communities	13
		Mainland Communities	11

## Data Availability

The data that support the findings of this study are available from Christine Muhumuza, but restrictions apply to the availability of these data, which were used under license for the current study, and so are not publicly available. Data are, however, available from the authors upon reasonable request and with permission of Christine Muhumuza.

## Abbreviations

AGYW: Adolescent Girls and Young Women
ART: Antiretroviral Therapy
HIV: Human Immunodeficiency Virus
HTS: HIV Testing Services
IDIs: In-depth Interviews
RCCS: Rakai Community Cohort Study
WHO: World Health Organization
