# Supplementary material for: Barriers, Facilitators and Preferences for HIV Testing Services among Adolescent Girls (15–19) and Young Women (20–24) in Rakai District, Central Uganda
Source: Res Sq. 2025 Jul 14:rs.3.rs-6744863. Preprint. [Version 1] doi: 10.21203/rs.3.rs-6744863/v1 (PMC12288541; doi:10.21203/rs.3.rs-6744863/v1)
Supplement: 1 [file NIHPPrs6744863v1-supplement-1.pdf]

Supplementary Files

This is a list of supplementary files associated with this preprint. Click to download.

- IDIGuideEnglishChristine.pdf
